# The epidemiology and treatment of femur fractures at a northern tanzanian referral centre

**DOI:** 10.11604/pamj.2015.22.338.8074

**Published:** 2015-12-04

**Authors:** Alexander Conor Hollis, Samuel Robert Ebbs, Faiton Ndesanjo Mandari

**Affiliations:** 1 College of Medical and Dental Sciences, University of Birmingham, Edgbaston, Birmingham, B15 2TT, United Kingdom; 2 Kilimanjaro Christian Medical Centre, PO Box 3010, Moshi, Tanzania

**Keywords:** Femoral fractures, orthopaedics, Tanzania, epidemiology

## Abstract

**Introduction:**

Femoral fractures are the most common presenting injury at the orthopaedic department in a large Tanzanian hospital. To date, there has been no current examination of the epidemiology of femoral fractures and the disease burden has not been quantified.

**Methods:**

A retrospective descriptive study of patient records in the orthopaedic department at Kilimanjaro Christian Medical Centre (KCMC) was performed. Patient demographics, aetiology of fractures, diagnosis and treatment were all recorded.

**Results:**

A total of 540 consecutive patient admission records were reviewed over a 9 month period. Of these 540 cases, 213 (39%) were diagnosed with a femoral fracture. The 21-30 age group were the most commonly affected by femur fractures (20% n = 42). Within this group, motor traffic accidents (MTA) were the cause of 71% of injuries (n = 30). For males, MTA's caused 59% of all femur fractures (n = 80), while falls were the most common cause of femur fractures in females (70%; n = 49). 80% of the fractures in the 51-100 age group were caused by falls (n = 52). In both the male and female groups the most common fracture seen was mid shaft femoral fracture (males 33% (n = 48), females 25% (n = 18)). The most common treatment was skeletal traction used in 40% (n = 85) of patients.

**Conclusion:**

Femur fracture most commonly presented in males under age 30. Femur fracture was most commonly cause by MTAs in males and by falls in females. The most common diagnosis was mid shaft of femur fracture. Skeletal traction was the most frequent treatment.

## Introduction

There is a paucity of literature on the causes and epidemiology of fractures in East Africa, particularly fractures of the lower limb, most notably the femur. Schaftenaar et al conducted an epidemiological study of maxillofacial trauma in Dar ès Salaam [1]. There is also research by Casey et al [2] regarding traumatic injuries presenting to an emergency department in East Africa. They found despite the numbers affected by these traumatic injuries, the huge economic cost in developing countries remains significantly under-reported [2]. However none of these studies look at femoral fractures or even lower limb fractures as their primary outcome. We report our experiences of femoral fractures at Kilimanjaro Christian Medical Centre (KCMC), a large tertiary referral centre in the North of Tanzania. KCMC was opened in 1971 by the Good Samaritan Foundation and has grown over the years and now has 630 inpatient beds, serving a catchment population of over 15 million people. Its clinical services are diverse and it is recognised as one of the leading hospitals in Tanzania. In the UK, the most common cause of femoral fractures is osteoporotic falls in the elderly [3]. At the time of writing, there is no Tanzanian national data to facilitate a comparison. This illustrates the need for epidemiological studies of femoral fractures in East Africa as average life expectancy continues to increase. In 2014, fractures of the femur were the most frequent presentation to the orthopaedic department at KCMC, with open fractures of the lower limb also responsible for the second highest mortality rate in the department [4]. Road traffic accidents are one of the top five causes of death in Tanzania. They are the commonest cause of death in men in their twenties, and contribute significantly to the mortality rates in young children. Road traffic injuries and deaths are a growing threat to health of Tanzanian nationals and represent a significant public health concern [5]. There have been a number of reports demonstrating the importance of road traffic injuries in the aetiology of traumatic fractures in Tanzania, particularly in relation to maxillofacial trauma [1]. In Tanzania, the highest number of trauma injuries affects young men [1]. However, there is no available evidence regarding the pattern and aetiology of fractures in older age groups and in females. This study aims to fill this evidence gap, and presents the disease burden and most common causes of injury, so that preventative measures can be taken. This study aims to identify the aetiology, pattern of femoral fractures and their management in a consecutive series of patients attending KCMC in Northern Tanzania.

## Methods

This study was carried out in the Orthopaedic Department of the Kilimanjaro Christian Medical Centre (KCMC) in Moshi, Northern Tanzania. Approval for study was granted by both our University and the Head of the Orthopaedic Department at KCMC. Consecutive admissions were reviewed retrospectively from September 2014 through to May 2015, providing us with a 9 month data collection period. All data was extracted from contemporaneously maintained inpatient record books kept in the orthopaedic department. All femur fractures recorded during the study period were included in the study, with no exclusion criteria. We gathered demographic information including age, gender, type and aetiology of fracture and subsequent treatment. The diagnosis of the patient was determined by x ray findings that were reviewed and confirmed by senior members of the Orthopaedic Department. Aetiology of the fracture was assessed by the study team after reviewing the clinical history recorded by the clerking intern. Finally, treatment was defined by the study team as the documented course of action by the clerking intern. We aimed to identify the pattern of fractures by recording the aetiology according to age and gender. We also assessed the diagnosis of fracture according to gender. Finally we examined the treatment according to diagnosis.

## Results

**Figure 1:**
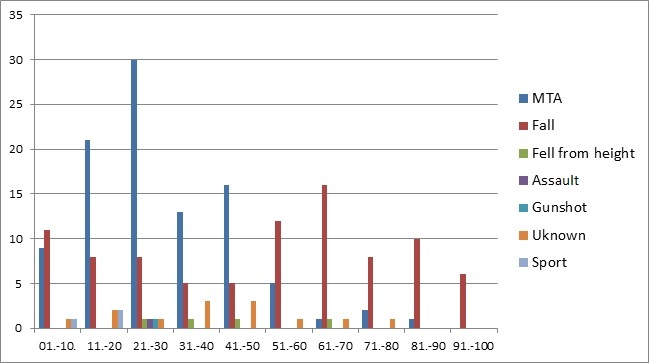
Aetiology of fracture according to age

**Figure 2:**
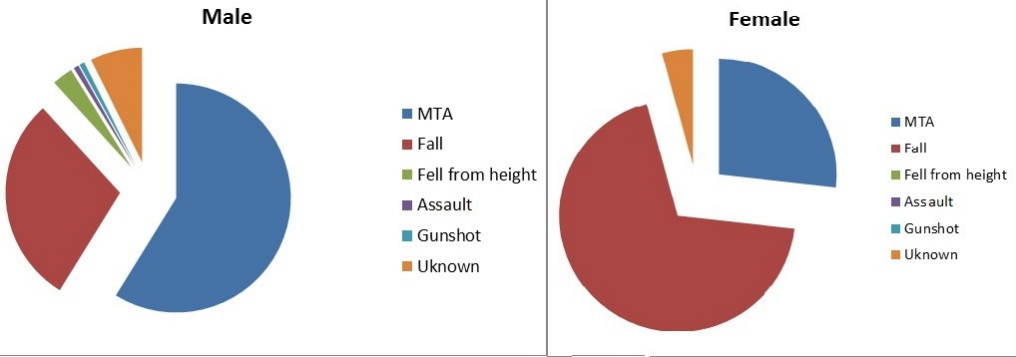
Aetiology of fractures according to gender

**Figure 3:**
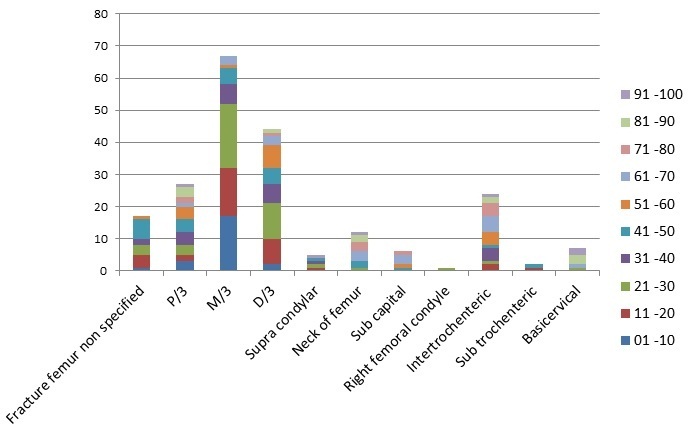
Pattern of fracture according to age

**Figure 4:**
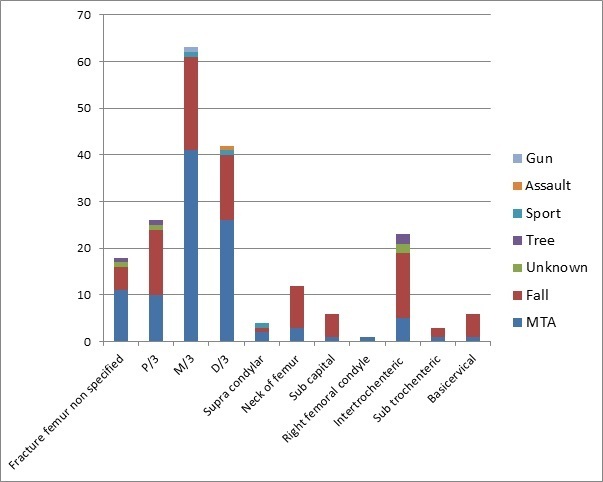
Pattern of fracture according to aetiology

A total of 540 patients were admitted to the orthopaedic department over a nine month period, September 2014 to May 2015. Of these 540 cases 213 patients (39%) were diagnosed with a femoral fracture. Of the 213 patients with fractured femur, 65% (N= 138) were males, and 34% (n = 72) were females. In 3 patients gender was not documented. In males, the most common group affected was 21-30 years (25%; n = 35), and in females it was age 51-60 years (17%; n = 12). Patients with femoral fractures ranged from 1 to 96 years old with a mean age of 39.2 years. The 21-30 age group were the most affected by femur fractures (n = 42). Within this group, motor traffic accidents (MTA's) caused the majority of injuries (71%; n = 30). Across all age groups, 49% (n = 102) of femoral fractures were caused by MTA's and 42% (n = 89) by falls. Falls were the most common cause of femoral fracture in the 51-100 age group (80%; n = 52) while fractures in those under age 50 were most commonly caused by MTA's. Interestingly within the MTA group, motor bike riders accounted for 54% (n = 55) of those with femoral fractures, while car drivers/passengers and pedestrians accounted for 42% (n = 43) and 4% (n = 4) of femoral fractures respectively (Figure 1). For males, MTA's were the largest cause of femur fractures (59%; n = 80) with falls being the second largest cause (29%; n = 40). In females, falls were the main cause 70% (n = 49) with MTA's being the second most common 27% (n = 19) (Figure 2). As mentioned previously, the most commonly affected age group was the 21-30 year olds (n = 42). Fractured mid shaft of femur was the most common diagnosis in this age group (48%; n = 20). Overall, fractured mid shaft of femur was the diagnosis in 31% (n = 67) of patients (Figure 3). In both males and females, the most common diagnosis was mid-shaft femoral fracture; in 33% (n = 48) of men and 25% (n = 18) of women. The second and third most common fractures in males were distal third (n = 30) and proximal third and neck of femur (both n = 16). In females the second and third most common fractures were distal third (n = 17) and proximal third (n = 10) respectively. By far the two most common causes of femoral fractures are MTAs and falls. These two causes combined accounted for 90% (MTA's; n = 102 and falls; n = 89l) of all femoral fractures. The most common fracture caused by MTA was mid shaft femur fracture (40%; (n = 41) followed by distal third femur fracture (25%; n = 26)). For falls, the most common fractures site was mid-shaft (22%; n = 20). Distal third, proximal third and intertrochanteric accounted together for 16% (n = 14) (Figure 4). There were 25 different treatment options recorded for the 213 patients. The most common treatment was skeletal traction (40%; n = 85). The most common injury treated by traction was mid shaft of femur fracture (39%; n = 33). The second and third most common treatments documented were ‘discussion with senior’ 9% (n = 20) and ‘treatment not recorded’ 9% (n = 19).

## Discussion

We found that road traffic accidents and falls were the most common causes of femoral fractures in patients attending a large regional centre. Accidents most commonly affected males under 30 years and motor bike riders. Much work has been done recently to improve the safety of the roads in Tanzania through the efforts of the World Health Organisation (WHO) and also through collaboration with the UK Government. However, there is still much work to do in this area. Road traffic accidents are still one of the largest causes of mortality in young men and laws on seatbelts and speed limits are often poorly enforced [6]. A femoral fracture is a severely debilitating injury for a patient. This is especially the case in a resource poor country such as Tanzania where treatment options are limited. The overwhelming use of traction as a treatment option highlights the chronic lack of resources for treatment of these injuries. Therefore, there is a two phased approach that is needed to help combat these injuries. First, improved primary prevention at a governmental level is needed including education and legislation to reduce the incidence of road traffic accidents. Second, there is a need for increased investment into both national and local healthcare initiatives so that the appropriate clinical equipment can be sourced to deal with these injuries. For instance, the most common fracture seen in the orthopaedic department was a mid-shaft femoral fracture. This is usually treated in KCMC with a Surgical Implant Generation Network (SIGN) nail. However, the hospital is reliant on these nails being imported from abroad. This means that often patients are not able to undergo surgery and therefore the only option available is treatment by skeletal traction. Another key issue highlighted by this research is the wide variation in treatments used for similar injuries. For 11 different fracture diagnoses there were 25 different treatments with seemingly little consistency in their application. This illustrates the need for the development of national guidelines and standards. Hopefully, this will lead to more consistent management strategies and as a consequence less variation in outcomes. This study has a number of limitations. The accuracy of the collected data is subject to the quality of the patient notes which have no recognised framework or agreed standard for completion. As a result, if clinical data was missing on certain variables, this was omitted from the final outcomes. This is an inherent limitation of retrospective case record studies. Outcome at follow-up was one of the variables that was rarely documented in the notes. Hence, there is need for a prospective study to assess the long-term consequences of current treatments available for femoral fractures at KCMC. However, despite these limitations the study provides a valuable baseline for further research. One of the key findings from the literature review is the paucity of epidemiological data in this area. Hopefully, this can be the first step in introducing a culture of systematic routine data collection to support audit and epidemiological studies of orthopaedic injuries in Tanzania. What literature that does exist only covers emergency medicine, spinal and maxillofacial fractures [1, 2, 7].

This topic is relevant to a global audience and by drawing on the experiences of other countries the developmental progress in this area can be accelerated. Countries such as the UK, with established public health programmes have collected epidemiological data over years in order to identify the population risks and healthcare needs in order to plan preventative interventions and hospital services. Public health drives, such as the enforcement of seatbelt wearing [8] are a vivid example of how epidemiological data can identify causes and lead to public policy changes that improve health outcomes in a country and reduce the burden of disease. It is also crucial for any potential investors, charitable organisations or government legislators to have a complete understanding of the nature and depth of the problem in Tanzania so they may target investment to tackle the underlying causes of injuries and gaps in healthcare provision. Our data also highlights falls as the largest cause of femoral fractures in patients over 50 years of age and in females. Falls have been researched in depth in the UK but needs further investigation in Tanzania. Co morbidities such as osteoporosis or vitamin d deficiency need to be investigated to determine any potential mediating role in fractures associated with falls. A crucial point is the ability of the surgeons at KCMC to appropriately treat those fractures following falls, in particular fractured neck of femur. In the UK, a patient with a fractured neck of femur is either treated with a dynamic hip screw (DHS), hemi-arthroplasty or a total hip replacement (THR) depending on the site of the fracture [9], while in Tanzania access to these specialised treatments is limited by the lack of available equipment. Austin Moor hemi-arthroplasty is the only available replacement option and there is a severe lack of dynamic hip screws and limited imaging options. There are also no facilities or expertise to perform a THR at KCMC. Specific research needs to be undertaken as this resource poor countries begin to experience greater life expectancy and a population age profile that more closely matches that of a western country. Currently, there are very limited treatment options in Tanzania for femur fractures following falls, and addressing this emerging health need requires effective international collaboration. Finally, further research is needed into the long-term outcomes of patients with femoral fractures in Tanzania, particularly with reference to their morbidity, mortality and quality of life.

## Conclusion

Males under 30 were the most commonly affected group with femur fractures. Road traffic accidents were the largest cause of fractures in males with falls accounting for most fractures in females. The most common diagnosis in both groups was mid shaft of femur fracture. Skeletal traction was the most commonly used treatment for femoral fractures. The implications from our findings are that there are two population groups that cause a disproportionately high burden of disease from seemingly preventable causes. Future research is needed to explore this high risk population and examine public health measures to reduce the incidence of such injuries.

## References

[1] Schaftenaar E, Bastiaens GJH, Simon ENM, Merkx MAW (2009). Presentation and management of maxillofacial trauma in Dar es Salaam, Tanzania. East Afr Med J..

[2] Casey ER, Muro F, Thielman NM, Maya E, Ossmann EW, Hocker MB, Gerardo CJ (2012). Analysis of traumatic injuries presenting to a referral hospital emergency department in Moshi, Tanzania. Int J Emerg Med..

[3] Van Staa TP, Dennison EM, Leufkens HGM, Cooper C (2001). Epidemiology of fractures in England and Wales. Bone..

[4] Kilimanjaro Christian Medical Centre website (2015). http://www.kcmc.ac.tz/.

[5] World Health Organisation (WHO) (2007). Road safety country profiles Tanzania.

[6] British High Commission, Dar es Salaam (2013). Road Safety Roundtable.

[7] Mhina R, Kinasha A, Kinuda SM (1993). The Aetiology, pattern and prognosis of fractures of the spine in Dar es Salaam, Tanzania. Central Afr J Med..

[8] Think Direct Gov UK (2015). http://think.direct.gov.uk/seat-belts.html.

[9] National Institute for Clinical Excellence (NICE) (2011). NICE guidelines on the management of hip fracture in adults.

